# Polymorphisms of *SP110* Are Associated with both Pulmonary and Extra-Pulmonary Tuberculosis among the Vietnamese

**DOI:** 10.1371/journal.pone.0099496

**Published:** 2014-07-09

**Authors:** Gregory J. Fox, Dinh Ngoc Sy, Nguyen Viet Nhung, Bing Yu, Magda K. Ellis, Nguyen Van Hung, Nguyen Kim Cuong, Luu Thi Lien, Guy B. Marks, Bernadette M. Saunders, Warwick J. Britton

**Affiliations:** 1 Respiratory and Environmental Epidemiology Research Group, Woolcock Institute of Medical Research, Glebe, Sydney, Australia; 2 Sydney Medical School, University of Sydney, Sydney, Australia; 3 Tuberculosis Research Group, Centenary Institute, Newtown, Sydney, Australia; 4 National Lung Hospital, Ba Dinh, Hanoi, Vietnam; 5 Hanoi Medical University, Hanoi, Vietnam; 6 Department of Medical Genomics, Royal Prince Alfred Hospital, Camperdown, Sydney, Australia; 7 Hanoi Lung Hospital, Hanoi, Vietnam; 8 Liverpool Hospital, Liverpool, Sydney, Australia; Institut Pasteur, France

## Abstract

**Background:**

Tuberculosis (TB) is an infectious disease that remains a major cause of morbidity and mortality worldwide, yet the reasons why only 10% of people infected with *Mycobacterium tuberculosis* go on to develop clinical disease are poorly understood. Genetically determined variation in the host immune response is one factor influencing the response to *M. tuberculosis*. SP110 is an interferon-responsive nuclear body protein with critical roles in cell cycling, apoptosis and immunity to infection. However association studies of the gene with clinical TB in different populations have produced conflicting results.

**Methods:**

To examine the importance of the *SP110* gene in immunity to TB in the Vietnamese we conducted a case-control genetic association study of 24 *SP110* variants, in 663 patients with microbiologically proven TB and 566 unaffected control subjects from three tertiary hospitals in northern Vietnam.

**Results:**

Five SNPs within *SP110* were associated with all forms of TB, including four SNPs at the C terminus (rs10208770, rs10498244, rs16826860, rs11678451) under a dominant model and one SNP under a recessive model, rs7601176. Two of these SNPs were associated with pulmonary TB (rs10208770 and rs16826860) and one with extra-pulmonary TB (rs10498244).

**Conclusion:**

*SP110* variants were associated with increased susceptibility to both pulmonary and extra-pulmonary TB in the Vietnamese. Genetic variants in *SP110* may influence macrophage signaling responses and apoptosis during *M. tuberculosis* infection, however further research is required to establish the mechanism by which *SP110* influences immunity to tuberculosis infection.

## Introduction

Tuberculosis (TB) is an airborne infectious disease that remains a major global health priority [1], with 8.7 million new cases and 1.4 million deaths each year [2]. Although one third of the world's population has been infected with *M. tuberculosis*, the causative bacterium [3], less than 10% of infected individuals will develop active disease in their lifetimes [4]. The probability that an infected individual develops TB depends upon the capacity of the host cellular immune system to recognize and control the infection [5]. The effectiveness of the immune response is influenced by the interplay between a range of environmental [6] and genetic factors [7].

Genetic variability is an important determinant of the effectiveness of the host immune response to *M. tuberculosis*. Studies in twins [5], genetic linkage studies, association studies [8] and genome wide association studies [9], [10] have demonstrated that host genetic factors modulate the risk infected individuals will develop TB [7]. However, there is heterogeneity in the pattern of genetic associations with TB in different populations [7]. This heterogeneity between published studies is likely to be explained in part by genetic variation between the populations and gene-environment interactions [5], underlining the importance of undertaking association studies in a range of different settings.

The *SP110* gene was first implicated in the genetic regulation of the immune response to *M. tuberculosis* in a study of its murine homolog, the interferon-inducible nuclear protein gene 1 (*Ipr1*) in hypersusceptible mice [11]. Mice with the disease-causing variant of the gene developed necrotic pulmonary cavities similar to those typical of human pulmonary TB [12]. The gene's effect was to modulate macrophage function, independent of Th1 cytokine-producing T-lymphocytes, or inducible nitrous oxide synthase [13]. Despite the fact that *SP110* is interferon inducible, the increased susceptibility of this mouse variant was IFN-γ independent. SP110 protein is considered to affect mycobacterial immunity by influencing cell differentiation, activation and apoptosis [14], although the mechanism for this effect remains incompletely understood.

In humans, *SP110* variants have been implicated in a number of diseases including hepatic veno-occlusive disease with immunodeficiency (VODI) [15], [16], viral hepatitis infection [17] and TB [18]–[23]. Previous studies of the gene in human TB have found apparently inconsistent results. Two single nucleotide polymorphisms (SNPs) of *SP110* (rs3948464 and rs2114592) were found to be associated with TB in populations from West Africa [18], while significant associations were identified in India (rs1427294) [24] and China (rs1135791) [25]. However, studies of the gene in other settings have found no evidence of an association [19]–[23] A recent meta-analyses of five SNPs found no consistent associations with TB [26]. Given the heterogeneity of these results, further studies in Asian populations are needed to clarify the relationship between genetic variation in *SP110* and TB. Consequently, this study investigated whether SNPs of *SP110* were associated with TB in a Vietnamese population.

## Materials and Methods

### Ethics statement

Ethics approval was obtained from the Human Research Ethics Committee at the University of Sydney, the Scientific Committee at the National Lung Hospital and the Institutional Review Board of the Ministry of Health, Vietnam. Research participants provided written informed consent.

### Subjects

Vietnamese patients with TB were recruited from inpatients at three lung disease hospitals in northern Vietnam between 2009 and 2012. Patients with pulmonary TB were sputum smear positive for acid fast bacilli (AFB) diagnosed using standard criteria [27] (Table S1). Patients with extra-pulmonary tuberculosis were diagnosed using usual standardized clinical criteria, listed in the Table S2. Patients were recruited as cases within seven days of commencing treatment. HIV serology was performed using commercial antigen detection kits (Alere Inc, MA, USA). Cases were excluded if they were HIV positive or had a known history of HIV. One sputum sample from each case with pulmonary disease was cultured using either solid or liquid culture media. Sputum smear was performed using fluorescent microscopy. Subjects with both confirmed pulmonary TB and extra-pulmonary TB were included in the pulmonary TB group. Controls were health care workers and medical students with no history of TB and normal chest radiographs, recruited from the three lung disease hospitals and their affiliated district clinics. Two trained radiologists read the chest radiographs for patients and control subjects independently to exclude active disease in controls. Participants gave three to five milliliters of peripheral blood. Genomic DNA was extracted from peripheral blood leukocytes using Qiagen kits according to manufacturer's instructions (Qiagen Corp, CA).

### Selection of SNPs

Tag SNPs were selected across the *SP110* gene using HapMap data (http://hapmap.ncbi.nlm.nih.gov/) and the International HapMap Project (http://www.hapmap.org/). Data from HapMap Phase III were obtained from the Han Chinese population (CHB), applying the Tagger algorithm to cover 100% of alleles with a pair-wise r^2^ linkage disequilibrium cut-off of 0.8 [28]. The software was used to force the inclusion of selected SNPs that had been associated with TB in previously published studies [18], [24], [25]. Selection upon data for the Han Chinese Beijing (HCB) population was based upon their genetic similarity with Vietnam's majority Kinh ethnic group [29].

### Genotyping

PCR primers were designed in multiplex reactions for 24 SNPs and were allocated to three separate assays. Primer sequences have been included in the Table S3. Multiplex PCR and primer extension were performed according to standard protocols [30]. Multiplex PCR was undertaken in 5 µL reactions in 384 well plates, using approximately 10 ng of genomic DNA, 0.5 µL of 10X proprietary PCR buffer (Sequenom Corporation, San Diego, CA), 20 nmol MgCl_2_, 2.5 nmol of each dNTP, 0.5 pmol of the primer mix, 1.0 U *Taq* DNA polymerase (Roche Applied Science, Germany) in TAE (40 mM Tris, 0.01 mM acetic acid, 1 mM EDTA) and 0.8 µL double distilled water. PCR thermal cycling was performed in a PCR thermocycler, comprising initial denaturation of 2 min, then 45 cycles of denaturation at 94°C for 30 seconds, annealing at 56°C for 30 seconds, and extension at 72°C for 1 minute, then a final extension at 72°C for 1 minute. Unincorporated deoxyribonucleoside triphosphates were dephosphorylated by adding 0.5 U shrimp alkaline phosphatase (SAP) enzyme, 0.17 µL SAP Buffer (10x), and 1.53 µL of PCR grade water to each well. The plates were gently vortexed and centrifuged at 2000 g for 30 seconds before incubating them at 37°C for 40 minutes, and transferring them to an 85°C incubator for 5 minutes to denature the SAP enzyme. Primer extension was performed after adding to reaction 0.2 µL iPLEX termination mix, 0.04 µL iPLEX enzyme (Sequenom Corporation, CA, USA), 4.7 to 14.1 nmol extension primers, 0.2 µL buffer mix (10x) and 0.62 µL double distilled water. The mixture was gently mixed and centrifuged for 30 s at 1000 rpm. Initial denaturation was 30 seconds at 94°C. 40 cycles comprised denaturation at 94°C followed by five looped cycles, each with annealing at 52°C for 5 seconds and then extension at 80°C for 5 seconds. A final extension was performed 72°C for 3 minutes, and the samples were then cooled to 4°C. Then 16 µL of water was added to each well. A pre-treated cationic resin (Sequenom Corporation, San Diego) was subsequently added to each mixture of reaction products to remove salts, and the plates were centrifuged at 3200 g for 5 minutes.

SNPs were typed using the Matrix Assisted Laser Desorption/Ionisation Time-Of-Flight (MALDI-TOF) mass spectrometry, and spectral peaks were analyzed with Typerv4.0 software (Sequenom) [30].

### Statistical and bioinformatics analyses

Alleles for all tag SNPs were tested for an association with all forms of TB using Fisher's exact test. Adjusted analysis was conducted for SNPs that had p-values below a cut-off of 0.1 in the allelic analysis and were polymorphic. If age and gender were found on univariate analysis to be associated with TB, they were to be incorporated as covariates in multivariate analysis. In the adjusted analysis, both recessive and dominant models of inheritance were applied using multivariate analysis for all TB, pulmonary TB and extra-pulmonary TB. To account for multiple testing, a corrected p-value threshold was determined based upon analysis of the effective number of independent tests performed for all SNPs tested in the first stage analysis, excluding SNPs that were non-polymorphic. Following the methods described by Nyholt [31] and Li and Ji [32], we applied the SNPSpD algorithm to determine the effective number of independent tests:
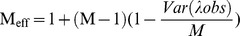



Where M_eff_ is the effective number of variables, Var(λ_obs_) is the ratio of the observed eigenvalue variance, and M is its maximum ratio. Using this method, the effective number of independent marker loci, M_eff_, was calculated to be 6.4507, defining an experiment-wide significance threshold of 0.007751 for each SNP to achieve an experiment-wide overall alpha level of 0.05.

The exact Hardy Weinberg Equilibrium (HWE) statistic was used to test deviation from HWE among controls. The χ^2^ test was performed to compare allele frequencies between our study population and the Han Chinese (CHB) population, using data from available public databases (http://www.ncbi.nlm.nih.gov/SNP/). Multiple permutation testing was performed to identify haplotype blocks using Haploview (version 4.2) [28]. Linkage Disequilibrium (LD) plots were compiled using Haploview. Statistical analyses were conducted using SAS (v9.2, Cary Corp, NC, USA) [28], [33].

### In silico analyses

Multi-point imputation was used to test the associations identified and develop probability distributions of untyped genotypes using SNPTEST version 2 (https://mathgen.stats.ox.ac.uk/genetics_software/snptest/snptest.html).

*In silico* bioinformatic analyses to explore the predicted functional impacts of identified SNPs upon protein function were performed using SeattleSeq Annotation 134 (version 7.0.5, http://snp.gs.washington.edu/SeattleSeqAnnotation134/) and MutationTaster (http://mutationtaster.org) [34]. Grantham scores were calculated, where possible [35], [36].

For alleles found to be significantly associated with TB in our study we planned to perform a meta-analysis if there were published data from at least 5 studies in genetically similar ethnic groups. In the absence of sufficient numbers of studies from comparable populations, we planned to present the estimates of each study without presenting a summary estimate.

## Results

A total of 566 control subjects and 663 patients were recruited, including 530 patients with smear positive pulmonary TB and 133 patients with extra-pulmonary TB. The features of the study population are described in Table 1. Patients and controls differed in age and gender (p<0.001), and this was adjusted for in the multivariate analysis.

**Table 1:** Baseline characteristics of TB patients and controls.

	Cases (TB Patients)	Controls
Number	663	566
Mean age, years (SD)	44.6 (17.3)	30.4 (10.6)
Males (%)	485 (73.2%)	214 (37.8%)
Vietnamese born (%)	100%	100%
HIV negative (%)	618/638 (97%)^1^	
Pulmonary TB	530	
Extrapulmonary TB		
All forms	133	-
Lymph node	9	-
Meningeal	7	-
Pleural	105	-
Abdominal	10	-
Other	2	-

1 HIV serology results were not available for 25 TB patients included in the study. 19 of these stated they had no past history of HIV and had no features of HIV, and data was missing for one subject. SD: standard deviation.

**Figure 1:**
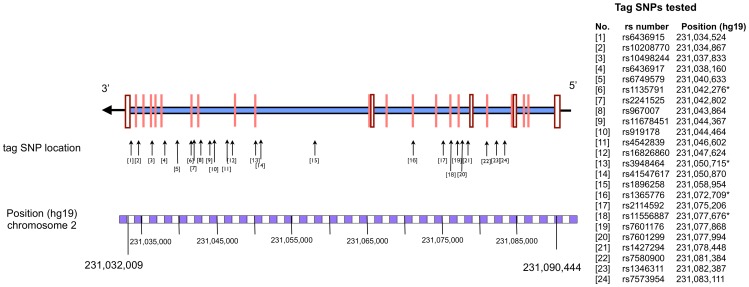
Human SP110 genomic structure showing the location of tested tag SNPs.

A total of 24 tag SNPs were selected, including four in the exonic regions and 20 in intronic regions. Schematic representations of the location of tag SNPs on the *SP110* gene are shown in Figure 1. Overall, 97.4% of included samples were “called” with moderate to high degree of confidence using standard algorithms with Typer v4.0 (Sequenom Inc, CA, USA) [30].

All genotyped SNPs were in Hardy Weinberg Equilibrium. Two of the 24 SNPs were non-polymorphic.

### Findings in all forms of tuberculosis

**Figure 2:**
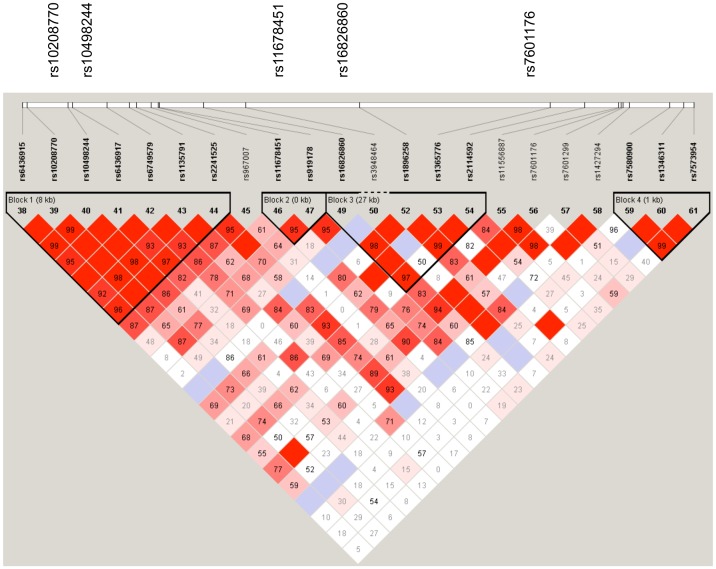
Linkage Disequilibrium Plot of SNPs of *SP110* with associations with tuberculosis. Pairwise D′ values are written as a percentage in each square. High D′ values are dark, low D′ values are light. SNPs associated with tuberculosis in multivariate analysis shown above the plot.

Comparison of allelic frequencies between cases and control subjects are shown in Table 2. The effective significance p-value threshold for the 22 polymorphic alleles tested was calculated to be 0.0078, which was applied to the second stage multivariate analysis. Table 3 shows the results of multivariate analysis, with unadjusted analyses shown in Table S4. After adjusting for age and gender in a multivariate analysis, four of these SNPs were associated with all forms of TB assuming a dominant inheritance model (rs10208770, rs10498244, rs11678451 and rs16826860) and one using a recessive model of inheritance (rs7601176), as shown in Table 3. Unadjusted analyses are shown in Table S4. Odds ratios were unable to be calculated for rs3948464 and rs1427294 using a dominant model, as there was a zero value in at least one cell of the two-by-two table. There were two associated SNPs in linkage disequilibrium block 1 (rs10208770, rs10497224), one in LD block 2 (rs11678451) and one was located in LD block 3 (rs16826860) as shown in Figure 2. Multiple permutation testing using Haploview (n = 10,000 variations) found no haplotype blocks or SNPs that met statistical significance. SNP rs10498244 had the highest permutation p-value (p = 0.0904).

**Table 2:** Allelic frequencies for all tuberculosis patients.

*SNP rs number*	*Alleles (Minor:Major)*		*Minor allele frequency (controls)*	*(%)*	*Major allele frequency (controls)*	*(%)*	*Minor Allele frequency (TB patients)*	*(%)*	*Major allele frequency (TB patients)*	*(%)*	*p value* *
**rs10208770**	G	T	125	(11%)	1007	(89%)	191	(14.5%)	1129	(85.5%)	0.013
**rs10498244**	C	T	165	(14.6%)	967	(85.4%)	247	(18.7%)	1071	(81.3%)	0.007
**rs1135791**	C	T	191	(16.9%)	941	(83.1%)	262	(19.8%)	1058	(80.2%)	0.06
**rs11678451**	C	T	165	(14.6%)	967	(85.4%)	237	(18%)	1081	(82%)	0.025
**rs16826860**	A	G	342	(30.2%)	790	(69.8%)	447	(33.9%)	871	(66.1%)	0.051
**rs3948464**	T	C	8	(0.7%)	1124	(99.3%)	1	(0.1%)	1317	(99.9%)	0.015
**rs7601176**	A	G	129	(11.4%)	1003	(88.6%)	115	(8.7%)	1203	(91.3%)	0.03
**rs1427294**	C	T	8	(0.7%)	1124	(99.3%)	2	(0.2%)	1316	(99.8%)	0.052

SNP: Single nucleotide polymorphism. Rs number  =  Reference Single Nucleotide Polymorphism number.

*Unadjusted P-value calculated using Fisher’s exact test. Results presented for SNPs included in adjusted analysis.

**Table 3:** Association of SNPs with pulmonary TB and extra-pulmonary TB by allelic analysis, by adjusted multivariate analysis.

SNP	Group	OR (95% CI) Dominant model^#^	p-value	OR (95% CI) Recessive model^#^	p-value
rs10208770		**GG+GT vs TT**		**GG vs GT+TT**	
	PTB	**1.65 (1.17–2.34)**	**0.005** *	3.01 (0.84–10.76)	0.090
	EPTB	1.49 (0.92–2.42)	0.106	5.33 (1.41–20.14)	0.014
	Pleural TB	1.36 (0.79–2.33)	0.266	**6.96 (1.82–26.58)**	**0.005**
	All TB	**1.64 (1.19–2.26)**	**0.002** *	4 (1.28–12.58)	0.017
rs10498244		**CC+CT vs TT**		**CC vs CT+TT**	
	PTB	1.52 (1.11–2.09)	0.010	2.44 (0.9–6.65)	0.081
	EPTB	1.42 (0.91–2.21)	0.122	**4.8 (1.68–13.72)**	**0.003** *
	Pleural TB	1.44 (0.89–2.34)	0.139	**5.49 (1.81–16.68)**	**0.003**
	All TB	**1.55 (1.16–2.07)**	**0.005** *	2.96 (1.23–7.09)	0.015
rs1135791		**CC+CT vs TT**		**CC vs CT+TT**	
	PTB	1.36 (1–1.85)	0.053	1.32 (0.57–3.06)	0.522
	EPTB	1.41 (0.91–2.17)	0.122	2.33 (0.89–6.11)	0.085
	Pleural TB	1.37 (0.85–2.2)	0.199	2.57 (0.92–7.18)	0.072
	All TB	1.4 (1.05–1.86)	0.021	1.61 (0.76–3.37)	0.211
rs11678451		**CC+CT vs TT**		**CC vs CT+TT**	
	PTB	1.4 (1.02–1.93)	0.038	1.13 (0.45–2.81)	0.796
	EPTB	1.72 (1.11–2.66)	0.014	1.4 (0.41–4.82)	0.594
	Pleural TB	1.81 (1.13–2.91)	0.014	1.8 (0.52–6.26)	0.354
	All TB	1.54 (1.15–2.06)	**0.004** *	1.16 (0.5–2.69)	0.722
rs16826860		**AA+AG vs GG**		**AA vs AG+GG**	
	PTB	1.5 (1.12–2.02)	**0.007** *	2.13 (1.33–3.43)	**0.002** *
	EPTB	1.2 (0.8–1.8)	0.373	0.79 (0.35–1.76)	0.565
	Pleural TB	1.21 (0.77–1.89)	0.401	0.74 (0.3–1.84)	0.523
	All TB	1.47 (1.12–1.92)	**0.005** *	1.74 (1.12–2.7)	0.014
rs3948464		**TT+TC vs CC**		**TT vs CT+CC**	
	PTB	na	na	6.17 (0.74–51.69)	0.093
	EPTB	na	na	na	na
	Pleural TB	na	na	na	na
	All TB	na	na	8.22 (0.99–68.42)	0.051
rs7601176		**GG+GA vs AA**		**GG vs GA+AA**	
	PTB	7.01 (1.09–45.04)	0.040	1.56 (1.07–2.25)	0.019
	EPTB	0.66 (0.15–2.93)	0.581	1.65 (0.95–2.86)	0.076
	Pleural TB	0.94 (0.17–5.29)	0.940	1.67 (0.9–3.11)	0.107
	All TB	2.21 (0.54–9.05)	0.268	1.64 (1.17–2.31)	**0.005** *
rs1427294		**TT+TC vs CC**		**TT vs TC+CC**	
	PTB	na	na	3.24 (0.65–16.17)	0.152
	EPTB	na	na	na	na
	Pleural TB	na	na	na	na
	All TB	na	na	4.32 (0.87–21.36)	0.073

Adj  =  Adjusted for age & gender. na  =  Not calculated as zero count in at least one cell of the two by two table. EPTB  =  extrapulmonary TB, All TB =  all forms of TB. Pleural TB is a sub-set of extrapulmonary TB.

*indicates SNPs for which the p-value was below the adjusted effective study-wide level of statistical significance (<0.00775), taking into account the number of effective tests [31].

### Findings in pulmonary TB

An association was identified between pulmonary TB and two SNPs, rs10208770 (OR 1.65, 95% CI 1.17–2.34, p_adj_ = 0.005), rs16826860 (OR 1.50, 95% CI 1.12–2.02, p_adj_ = 0.007), applying a dominant mode. Using a recessive model, an association was also shown with pulmonary TB for rs16826860 (OR 2.13, 95% CI 1.33–3.43). Allelic frequencies for pulmonary TB are included in Table S5.

### Findings in extra-pulmonary TB

Using recessive models there was a significant association between rs10498244 (OR 4.80, 95% CI 1.68–13.72, p = 0.003) and extrapulmonary TB. Both rs10498244 (OR 5.49, 95% CI 1.81–16.68, p = 0.0027) and rs10208770 (OR 6.96, 95% CI 1.82–26.58, p = 0.0046) were associated with pleural TB.

Predicted functional effects of the associated SNPs compiled using SeattleSeq and Mutation Taster are shown in Table 4. Figure 1 shows the tested SNPs and the linkage disequilibrium blocks for *SP110*. Schematic representations of the location of tag SNPs on the *SP110* gene are shown in Figure 1. The Linkage Disequilibrium plot in Figure 2 demonstrates that the associated SNPs lie primarily within three adjacent LD blocks.

**Table 4:** Summary of SNPs in adjusted analysis for all tuberculosis patients.

*rs number*	*Alleles (Minor, major)*		*Minor allele % among controls*	*Major allele % among controls*	*Minor allele % among TB patients*	*Major allele % among TB patients*	*p-value* *	Potential effect on protein^a^
rs10208770	G	T	11%	89%	14.5%	85.5%	0.013	No effect
rs10498244	C	T	14.6%	85.4%	18.7%	81.3%	0.007	No effect
rs1135791	C	T	16.9%	83.1%	19.8%	80.2%	0.06	Amino acid change M>T. Splice site changes. Possible downstream effect on bromo domain, Zn finger, SAND domain and nuclear localisation signal.
rs11678451	C	T	14.6%	85.4%	18%	82%	0.025	No effect
rs16826860	A	G	30.2%	69.8%	33.9%	66.1%	0.051	No effect
rs3948464	T	C	0.7%	99.3%	0.1%	99.9%	0.015	L>S. Splice site changes.
rs7601176	A	G	11.4%	88.6%	8.7%	91.3%	0.03	No effect
rs1427294	C	T	0.7%	99.3%	0.2%	99.8%	0.052	No effect.

SNP: Single nucleotide polymorphism. Rs number  =  Reference Single Nucleotide Polymorphism number.

a Predicted functional effect on protein synthesis.

*P-value for unadjusted allelic association between all TB patients and control subjects calculated using Fisher’s exact test. M  =  Methionine, T  =  Threonine, S =  serine, L =  leucine.

### Bioinformatic analysis

SeattleSeq was used to annotate the data and this revealed that among the associated SNPs, effects were predicted for rs3948464 (L>S, Leucine>Serine) and rs1135791 (M>T; Methionine>Threonine). This was confirmed with *in silico p*rediction of the functional impacts of associated polymorphisms using the MutationTaster algorithm (Table 4). Grantham scores, calculated by SeattleSeq, were available for rs1135791 (score 26) representing conservative chemical changes. Imputational analysis did not find any other SNPs in the regions near the associated SNPs to be more strongly linked to disease.

Two of the SNPs associated with TB in the present study had been previously shown to have an association with TB in other populations, rs3948464 (with an effect shown in the opposite direction [18]) and rs1135791 [37], [38]. It remains unclear whether rs3948464 itself has a functional effect. An explanation for the difference between African and Asian populations is likely to relate to the different population prevalences of the SNPs. Among Africans the allele frequency of the T allele is 14%, while only 0.5% of Chinese have this variant. Therefore it is likely that the studies in China and Vietnam are not powered to detect associations between this SNP and disease. However, it is significant that the Vietnamese, Chinese and West African studies have found the same LD block to be associated with TB susceptibility. This suggests that while the identified markers may not directly have a biological effect, the SNPs in LD block 1 may be linked to a nearby disease-causing SNP. Together, these studies support a role for SP110 polymorphisms in the pathogenesis of TB.

Forest plots showing results of meta-analyses of associated SNPs with other published studies are shown in the Figure S1, demonstrating heterogeneity between published studies, with a pooled analysis not reaching statistical significance. The predicted protein structure of SP110 is shown in Figure S3.

## Discussion

Speckled Protein 110 kD (SP110) is a component of the multi-protein complex associated with the promyelocytic leukemia protein nuclear body that regulates transcription and influences aspects of the macrophage lifecycle, including cell differentiation, activation and apoptosis [14]. This genetic association study has demonstrated the association between six SNPs of *SP110* and susceptibility to TB among the Vietnamese people, of which two remained associated with pulmonary disease (rs10208770, rs16826860 in the dominant model) and one with extra-pulmonary disease (rs10498244 in the recessive model).

This is the first study to show an association between *SP110* variants and both extrapulmonary and pulmonary disease, and the first published study of *SP110* in the Vietnamese. In this study, the SNP rs10498244 was associated with more than a four times greater odds of extrapulmonary TB in patients than controls in the recessive model. A second SNP on the same LD block (rs10208770 came close to reaching statistical significance (p = 0.014) using a recessive model, likely explained by being in linkage disequilibrium with a causative SNP. Both SNPs were associated with pleural TB, which was the cause of most extrapulmonary disease. We also tested our subjects for three SNPs that have been shown to be associated with extrapulmonary TB in an Indian population [24], however among our population of 133 Vietnamese patients with extra-pulmonary TB we found no association for any of them. The difference between the two studies may be explained by differences in the site of extrapulmonary disease. Most patients with extrapulmonary TB in our study had pleural TB, while all patients in the Indian study had presumed lymph node disease.

The finding of multiple SNPs that are associated with TB in this study is strongly supportive of a biological role for SP110 protein in the pathogenesis of the disease. This may either represent a single variant, or several variants. Given that several associated SNPs were in strong linkage disequilibrium with each other, this points towards a possible common causal SNP. Two of the SNPs most strongly associated with TB, rs10208770 and rs10498244 were in strong LD, with a D′ value of 99%. Furthermore, both SNPs were associated with pleural TB in a dominant model. Together, these findings suggest that a TB-susceptible DNA variant is present under this LD region, and that this may confer susceptibility to pleural and pulmonary TB.

There have been few functional studies that elucidate the mechanism by which *SP110* SNPs predispose to disease. This study has found two of the associated SNPs (rs11678451 and rs3948464) may have functional effects by testing the downstream effects of genetic changes using *in silico* analysis. Possible mechanisms for these effects include an alteration of transcript splice sites and alteration of protein sequence. These domains are conserved between some species and hence may play an important role in interferon-mediated cell replication and apoptosis [14]. There are three potentially important splice altering SNPs affecting the functional domain (NM_004509.3, NM_004510.3 and NM_080424.2), some of which maybe affected by our identified SNPs to alter the SP110 protein functional domains [16], [44]. Recently, another genetic variant in *Sp110* (rs9061, T allele) was also found to be associated with TB, and when this variant was combined with an allele in *MYBBP1A*, which is also associated with TB in Han Chinese, the combined haplotype significantly increased the risk of TB [38]. *MYBBP1A* encodes a transcription factor required for haemopoiesis, and MYBBP1A also binds to RelA and represses NFκB, a critical signaling component in the pathway of macrophage activation [39]. SP110 binds to *MYBBP1A* in murine cells, and this interaction is required for SP110-induced apoptosis [38]. Therefore genetic variants in SP110 may influence macrophage signaling responses and apoptosis during *M. tuberculosis* infection, however the precise mechanisms by which *SP110* SNPs rs11678451 and rs3948464 result in susceptibility to disease are yet to be established.

In order to compare our results to other studies, we tested seven SNPs that had been previously associated with TB in one or more published studies. Rs1135791 has been significantly associated with TB in two published studies among the Han Chinese where the T>C allele was protective [37], [38]. Although this SNP was not statistically significant after adjustment in the Vietnamese, the LD block containing the SNP was significantly associated with the disease. Possible reasons for the lack of association with rs1135791 in the Vietnamese include (i) differences in genetic susceptibility between ethnic groups, such as different linkage disequilibrium patterns, and (ii) inadequate power in the present study. The Figures S1 and S2 summarize the findings of the published studies of the relationship between tuberculosis and SNPs that were tested in our study.

False positive results arising from multiple testing are an important consideration for genetic association studies [40]. In order to account for this possibility, we calculated the ‘effective’ number of independent tests using an algorithm that assessed the LD between selected SNPs. The derived p-value threshold of 0.0078 was then applied to the results of the adjusted analysis. The overly conservative Bonferroni adjustment was not appropriate in this study, owing to the high degree of correlation (strong linkage disequilibrium) between the tested SNPs that increases the *a priori* probability of an association [31], [32], [41]. Further, there was a high degree of dependence between the results of recessive and dominant models [42]. As a result, we believe this approach to assessing statistical significance is appropriate.

An important strength of this study is the clear phenotypic definitions for patients and controls. The microbiological confirmation of the pulmonary TB enabled clear classification of this group of patients, and chest radiograph enabled us to exclude active disease in controls and prevent misclassification of subjects. In addition, our method of selecting control subjects from within TB hospitals is a considerable strength, as health care workers in Vietnam have twice the likelihood of infection compared to other occupations. In a study by Lien et al, conducted among health care workers in the same setting as the present study, the prevalence of latent TB infection was shown to be 66.3% [43]. On this basis, it is likely that a high proportion of our control group had been infected with *M. tuberculosis* but not developed the disease, suggesting a degree of protection against progression to TB. Hence, this group is more likely to differ from patients in relation to the genetic and immunological factors responsible for disease progression. A limitation of this study was the different gender and age distribution between the control and patient groups. This difference is unlikely to have adversely affected our results, as most of the identified associations persisted in the multivariate analysis.

In summary, this study has found six tag SNPs for *SP110* associated with all forms of TB and one SNP associated with extra-pulmonary TB. Two of these SNPs were in close linkage disequilibrium, in a block that is largely conserved in human populations. The findings support a role for *SP110* in both pulmonary TB and extra-pulmonary TB. Further work will be required to clarify the functional impact of these genetic variants in *SP110* on the response of human macrophages to infection.

## Supporting Information

Figure S1 **Forest plot of the association between pulmonary TB and rs3948464 T>C in published studies.**
(TIFF)

Figure S2 **Forest plot of the association between pulmonary TB and rs1135791 T>C in published studies.**
(TIFF)

Figure S3 **Model of predicted protein structure of SP110.** Hollow box represents the SP110 nuclear body protein (713 amino acids in length). Solid lines represent functional domains. Dashed line represents a putative acetyl lysine binding site.
(TIFF)

Table S1 **Inclusion and exclusion criteria for tuberculosis patients.**
(DOCX)

Table S2 **Definitions of extra-pulmonary tuberculosis.**
(DOCX)

Table S3 **Extension primers for tested SNPs.**
(DOCX)

Table S4 **Allelic frequencies for all tuberculosis patients compared to control subjects.**
(DOCX)

Table S5 **Allele frequencies for patients with pulmonary TB compared to control subjects.**
(DOCX)

## 

### Data availability statement

Genotyping data used in this study have been deposited in a publically accessible repository at the Centenary Institute, University of Sydney available at http://www.centenary.org.au/wp-content/uploads/2014/04/SP110-data-Vietnam-PLOS.xls.
