# Supplementary figures and images for: Polymorphisms of SP110 Are Associated with both Pulmonary and Extra-Pulmonary Tuberculosis among the Vietnamese
Source: PLoS One. 2014 Jul 9;9(7):e99496. doi: 10.1371/journal.pone.0099496 (PMC4090157; doi:10.1371/journal.pone.0099496)

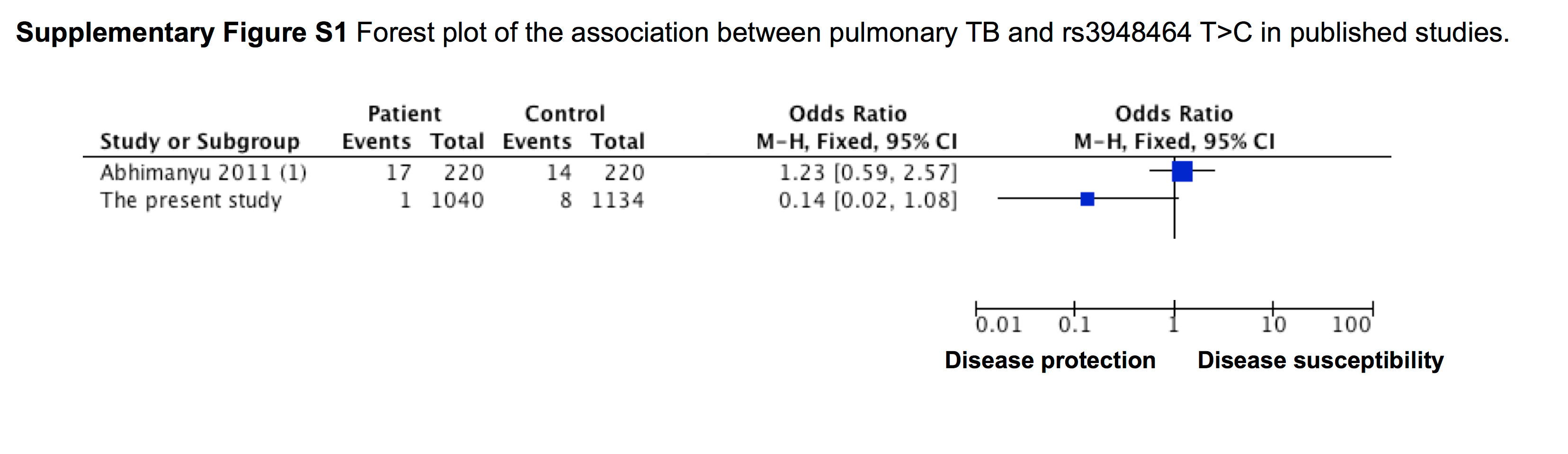

Supplement: Figure S1 — Forest plot of the association between pulmonary TB and rs3948464 T>C in published studies. (TIFF) [file pone.0099496.s001.tif]

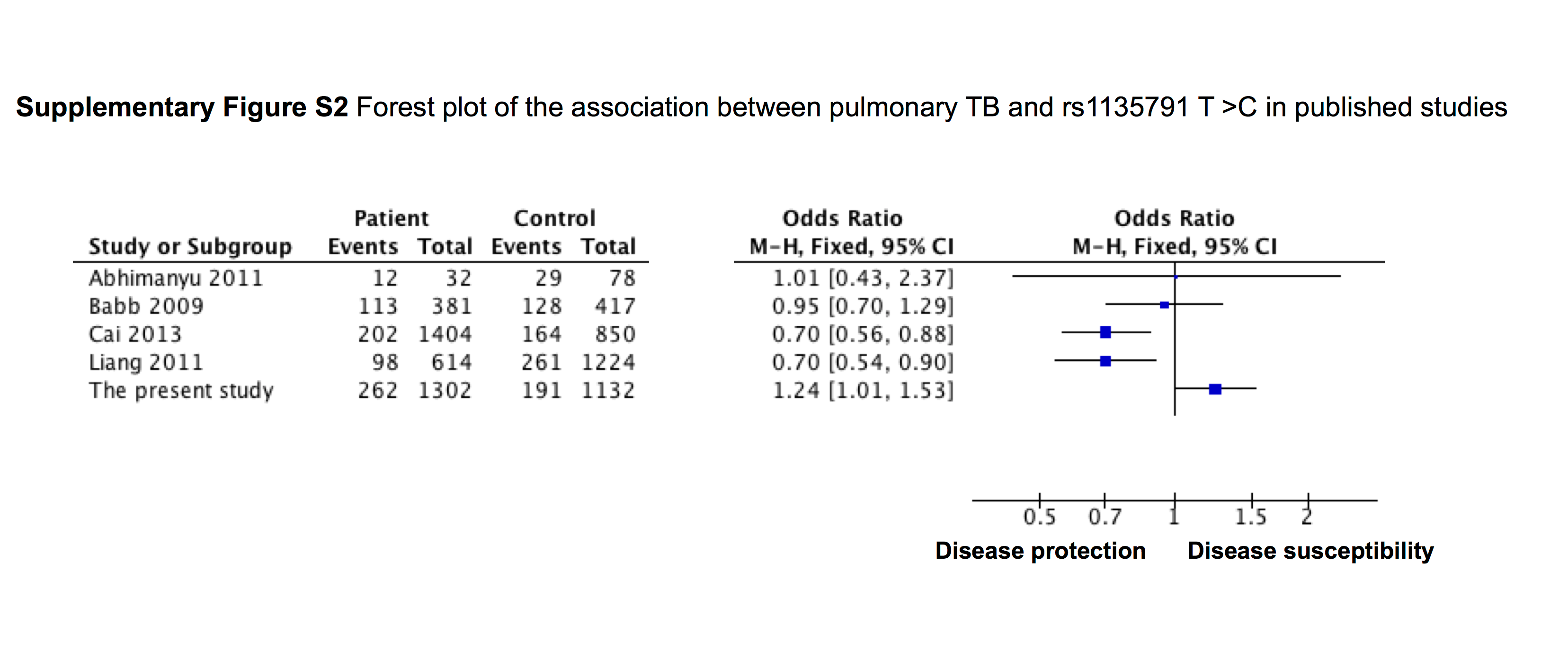

Supplement: Figure S2 — Forest plot of the association between pulmonary TB and rs1135791 T>C in published studies. (TIFF) [file pone.0099496.s002.tif]

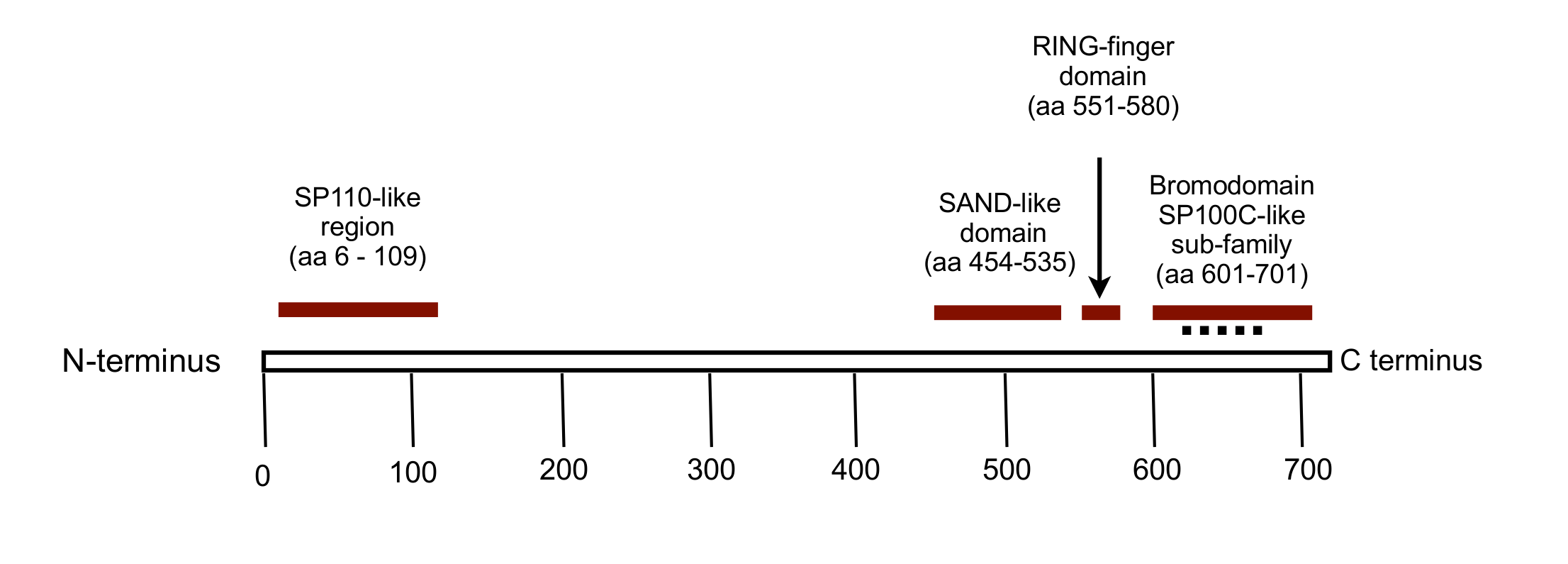

Supplement: Figure S3 — Model of predicted protein structure of SP110. Hollow box represents the SP110 nuclear body protein (713 amino acids in length). Solid lines represent functional domains. Dashed line represents a putative acetyl lysine binding site. (TIFF) [file pone.0099496.s003.tif]
