# Supplementary material for: Polymorphisms of SP110 Are Associated with both Pulmonary and Extra-Pulmonary Tuberculosis among the Vietnamese
Source: PLoS One. 2014 Jul 9;9(7):e99496. doi: 10.1371/journal.pone.0099496 (PMC4090157; doi:10.1371/journal.pone.0099496)
Supplement: Table S1 — Inclusion and exclusion criteria for tuberculosis patients. (DOCX) [file pone.0099496.s004.docx]

**Supplementary table S1: Inclusion and exclusion criteria for tuberculosis patients**

| **Inclusion criteria** | **Exclusion criteria** |
| --- | --- |
| AFB(+) positive pulmonary tuberculosis, that is either new or relapsed, and has one of the following:   - At least two AFB(+) specimens from two different sputum samples, OR - One AFB(+) specimen and X-ray changes consistent with tuberculosis, OR - One AFB (+) specimen with a positive culture | HIV positive test or history of HIV  Less than 18 years old |
