# Supplementary material for: Polymorphisms of SP110 Are Associated with both Pulmonary and Extra-Pulmonary Tuberculosis among the Vietnamese
Source: PLoS One. 2014 Jul 9;9(7):e99496. doi: 10.1371/journal.pone.0099496 (PMC4090157; doi:10.1371/journal.pone.0099496)
Supplement: Table S2 — Definitions of extra-pulmonary tuberculosis. (DOCX) [file pone.0099496.s005.docx]

**Supplementary Table S2: Definitions of extrapulmonary tuberculosis**

| **Site of Extra-pulmonary TB** | **Definition** |
| --- | --- |
| Pleural TB | Doctor diagnoses TB pleural effusion based on clinical findings and chest X-ray, and commences treatment for TB. Must include pleural fluid testing with at least 2 of the following:   - Straw coloured fluid - An exudate, with protein >5g/100mL - Positive smear of pleural fluid or pleura - Positive culture of pleural fluid or pleura - Positive pleural biopsy (on histology) |
| Meningeal TB | Include in study if subject meets both of the following criteria:   - Clinical meningitis (nuchal rigidity and abnormal CSF parameters) - CSF shows tuberculosis: either AFB (+) or culture positive   Collect blood when a positive result is available (culture or AFB). |
| Lymph node TB | Needle biopsy or surgical excision of a lymph node with positive smear or culture or histopathology.  At least one of these tests must be consistent with TB. |
| Other sites of extra-pulmonary TB | Positive histology, or smear or culture result from affected part of the body. |
