# Supplementary material for: Polymorphisms of SP110 Are Associated with both Pulmonary and Extra-Pulmonary Tuberculosis among the Vietnamese
Source: PLoS One. 2014 Jul 9;9(7):e99496. doi: 10.1371/journal.pone.0099496 (PMC4090157; doi:10.1371/journal.pone.0099496)
Supplement: Table S3 — Extension primers for tested SNPs. (DOCX) [file pone.0099496.s006.docx]

**Supplementary Table S3: Extension primers for tested SNPs**

| **dbS** | **First PCR Primer** | **Second PCR Primer** | **Unextended primer sequence** | **Allele 1** | **Mass of EP1** | **Allele 2** | **Mass of EP2** | **Allele 3** | **Mass of EP3** |
| --- | --- | --- | --- | --- | --- | --- | --- | --- | --- |
| rs2114592 | ACGTTGGATGGGGAATGTCTTTGCTGTATG | ACGTTGGATGAATAACCACAGATAGAGCAG | cctcTTGCTTTGACGTCTATGT | T | 6929.5 | C | 6945.5 | na | na |
| rs3948464 | ACGTTGGATGGCAAGAACTAAATGTGCCCG | ACGTTGGATGCCTCCTACATTGAGCTATTC | gagaAAATGTGCCCGAAAGTCCAGAT | C | 8284.4 | A | 8308.5 | G | 8324.5 |
| rs41547617 | ACGTTGGATGCTTGGAGCTTCTCTTGGATG | ACGTTGGATGAACATGAGAAGGGAGCTCAC | tCTGCCCCTGGACCAAA | G | 5362.5 | A | 5442.4 | na | na |
| rs10208770 | ACGTTGGATGGATTGCAGGATAGCTCAACC | ACGTTGGATGCTCAAACATCTGGAATTGCC | AAAGGAGGATGAAACCAACAT | G | 6792.5 | T | 6832.4 | na | na |
| rs10498244 | ACGTTGGATGCAGAGTGGATATCCAAACCC | ACGTTGGATGCAGACTCTCTGCTTGTTGTG | tgCTCCCTGGGGGAAAT | C | 5473.6 | T | 5553.5 | na | na |
| rs1135791 | ACGTTGGATGGTCACCTATCTGTCCCGTCA | ACGTTGGATGACGCAAAGAACTGGAAACGG | cCAGCTCTCCTAGGGTC | T | 5393.5 | C | 5409.5 | na | na |
| rs11556887 | ACGTTGGATGGTGAACAGCTTGGTTGAGGG | ACGTTGGATGTCCTACTTGAAGCCCCAACT | cccaaAGGGAGCTTCCTTCT | T | 6324.2 | C | 6340.2 | na | na |
| rs11678451 | ACGTTGGATGAAATTTGCTGCCGCAGGTTC | ACGTTGGATGCTGTGCTAATCTCCCTCAAG | agGGTGACTATGGACCACTA | C | 6413.2 | T | 6493.1 | na | na |
| rs1346311 | ACGTTGGATGGCTCTTCCACTCTCTAGTTG | ACGTTGGATGGACTGCACCTGTTTATACTG | aCACTCTCTAGTTGTGCAACC | C | 6588.3 | T | 6668.2 | na | na |
| rs1365776 | ACGTTGGATGTCTGGTCAACTCCAAAAAGG | ACGTTGGATGTCCAGCTTCCTCTTGTACTC | tctcaATAAGAAAAAAAGCCTCCCA | A | 7867.2 | G | 7883.2 | na | na |
| rs1427294 | ACGTTGGATGCTGAGCTTCAGTCATCTTCC | ACGTTGGATGTTTGGAAAGGGATTCAGAAC | TTCCGGGATCTTCAAC | C | 5079.3 | T | 5159.3 | na | na |
| rs16826860 | ACGTTGGATGAGACTGGTGCCATCTACAAG | ACGTTGGATGGGAATCTTGCTATGAAAACAG | AGAATAAGGAGCTTGTAAAC | G | 6445.3 | A | 6525.2 | na | na |
| rs1896258 | ACGTTGGATGAAAGGGCCTAAGATATGCCG | ACGTTGGATGGAGCTGGGACAAGATCTCT | gcgcGGGGATTCTCTTGGATTCT | A | 7348.8 | G | 7364.8 | na | na |
| rs2241525 | ACGTTGGATGCGTTTCTTCTGCAGTCTAGC | ACGTTGGATGTCCCTAGATGAAGGACTCAC | gTCCTGATCAATTACACACA | G | 6292.1 | A | 6372.1 | na | na |
| rs4542839 | ACGTTGGATGAACAACAGATGCTGGCAAGG | ACGTTGGATGTGGTTCTAGGTCTTTGAGGA | GTTTAACCCTTGTGAAAGA | C | 6074 | T | 6153.9 | na | na |
| rs6436915 | ACGTTGGATGCGAGTGGGCTTCCAAGAAAT | ACGTTGGATGGGGTTAGTTTTGATCATGGC | ccGGCTTCCAAGAAATGCATTTA | G | 7254.8 | T | 7278.8 | na | na |
| rs6436917 | ACGTTGGATGTGGCCCTAAAATGTTCCAC | ACGTTGGATGTTGTAAGTGCCAAAGGTAAC | gacTGTTCCACAGTGGGC | A | 5786.8 | G | 5802.8 | na | na |
| rs6749579 | ACGTTGGATGAATGCTGCAGCATGAAAAG | ACGTTGGATGTCATGGATGATCCTCTTGTC | cccctCAGCATGAAAAGCAAAAGAA | G | 7893.2 | T | 7917.2 | na | na |
| rs7573954 | ACGTTGGATGCAATCGCATAGGAAAACAAG | ACGTTGGATGCTGTTCTCTATCTTCCAAGAC | ggGCATAGGAAAACAAGAAAAGATG | C | 8077.3 | T | 8157.2 | na | na |
| rs7580900 | ACGTTGGATGGTCTCAAACCACAAACCACC | ACGTTGGATGACACCCTCTCTCCTAACTAC | TTTGGAAGGCCCAGCCC | T | 5442.6 | C | 5458.6 | na | na |
| rs7601176 | ACGTTGGATGAAGGGGCCACAAATGGATAA | ACGTTGGATGATGCCTTCCAATAGGATGGG | ccctATGACAATGAATCAGTTCTC | G | 7518.9 | A | 7598.8 | na | na |
| rs7601299 | ACGTTGGATGCTAAGCCCTGATCTAATACC | ACGTTGGATGCTGGGTACAAACCCAAACTC | gctcATACCAAAATGGTTTCACAC | A | 7552 | G | 7568 | na | na |
| rs919178 | ACGTTGGATGCTTGTGAATGTGTTTGGGAC | ACGTTGGATGCCCTTCTACAGTTCTCTGTC | TTGTGTTCCCTAAAGCTAT | A | 6040 | G | 6056 | na | na |
| rs967007 | ACGTTGGATGCTTCTTCGCCrCTGATACTG | ACGTTGGATGCCCTTGCCTCAAACAGTTAT | CCACTGATACTGTTCCTTC | C | 5936.9 | A | 5960.9 | na | na |

EP: Extension primer. PCR: Polymerase chain reaction.
