# Supplementary material for: Polymorphisms of SP110 Are Associated with both Pulmonary and Extra-Pulmonary Tuberculosis among the Vietnamese
Source: PLoS One. 2014 Jul 9;9(7):e99496. doi: 10.1371/journal.pone.0099496 (PMC4090157; doi:10.1371/journal.pone.0099496)
Supplement: Table S4 — Allelic frequencies for all tuberculosis patients compared to control subjects. (DOCX) [file pone.0099496.s007.docx]

**Supplementary table S4: Allelic frequencies for all tuberculosis patients compared to control subjects**

| *SNP*  *rs number* | *Alleles*  *(Minor:Major)* | | **Allelic frequencies in control subjects** | | | | **Allelic frequencies in all patients** | | | |  | |  |
| --- | --- | --- | --- | --- | --- | --- | --- | --- | --- | --- | --- | --- | --- |
|  |  |  | *Minor allele* | *(%)* | *Major*  *allele* | *(%)* | *Minor*  *allele* | *(%)* | *Major allele* | *(%)* | *p value** |  |  |
| rs10208770 | G | T | 125 | (11%) | 1007 | (89%) | 191 | (14.5%) | 1129 | (85.5%) | 0.013 |  |  |
| rs10498244 | C | T | 165 | (14.6%) | 967 | (85.4%) | 247 | (18.7%) | 1071 | (81.3%) | 0.007 |  |  |
| rs1135791 | C | T | 191 | (16.9%) | 941 | (83.1%) | 262 | (19.8%) | 1058 | (80.2%) | 0.06 |  |  |
| rs11556887 | T | C | 91 | (8%) | 1041 | (92%) | 98 | (7.4%) | 1220 | (92.6%) | 0.596 |  |  |
| rs11678451 | C | T | 165 | (14.6%) | 967 | (85.4%) | 237 | (18%) | 1081 | (82%) | 0.025 |  |  |
| rs1346311 | T | C | 93 | (8.2%) | 1039 | (91.8%) | 110 | (8.3%) | 1210 | (91.7%) | 0.942 |  |  |
| rs1365776 | G | A | 113 | (10%) | 1019 | (90%) | 129 | (9.8%) | 1191 | (90.2%) | 0.892 |  |  |
| rs1427294 | C | T | 8 | (0.7%) | 1124 | (99.3%) | 2 | (0.2%) | 1316 | (99.8%) | 0.052 |  |  |
| rs16826860 | A | G | 342 | (30.2%) | 790 | (69.8%) | 447 | (33.9%) | 871 | (66.1%) | 0.051 |  |  |
| rs1896258 | G | A | 253 | (22.3%) | 879 | (77.7%) | 266 | (20.2%) | 1054 | (79.8%) | 0.197 |  |  |
| rs2114592 | T | C | 241 | (21.3%) | 891 | (78.7%) | 261 | (19.8%) | 1057 | (80.2%) | 0.367 |  |  |
| rs2241525 | A | G | 236 | (20.8%) | 896 | (79.2%) | 245 | (18.6%) | 1073 | (81.4%) | 0.169 |  |  |
| rs3948464 | T | C | 8 | (0.7%) | 1124 | (99.3%) | 1 | (0.1%) | 1317 | (99.9%) | 0.015 |  |  |
| rs41547617 | A | C | 1132 | (100%) | 0 | (0%) | 1318 | (100%) | 0 | (0%) | na |  |  |
| rs4542839 | C | T | 760 | (100%) | 0 | (0%) | 88 | (100%) | 0 | (0%) | na |  |  |
| rs6436915 | T | G | 429 | (37.9%) | 703 | (62.1%) | 516 | (39.1%) | 804 | (60.9%) | 0.56 |  |  |
| rs6436917 | A | G | 554 | (48.9%) | 578 | (51.1%) | 633 | (48%) | 685 | (52%) | 0.656 |  |  |
| rs6749579 | G | T | 127 | (11.2%) | 1005 | (88.8%) | 140 | (10.6%) | 1178 | (89.4%) | 0.649 |  |  |
| rs7573954 | T | C | 323 | (28.5%) | 809 | (71.5%) | 373 | (28.3%) | 945 | (71.7%) | 0.928 |  |  |
| rs7580900 | C | T | 421 | (37.2%) | 711 | (62.8%) | 488 | (37%) | 832 | (63%) | 0.933 |  |  |
| rs7601176 | A | G | 129 | (11.4%) | 1003 | (88.6%) | 115 | (8.7%) | 1203 | (91.3%) | 0.03 |  |  |
| rs7601299 | A | G | 116 | (10.2%) | 1016 | (89.8%) | 139 | (10.5%) | 1179 | (89.5%) | 0.842 |  |  |
| rs919178 | G | A | 507 | (44.8%) | 625 | (55.2%) | 561 | (42.5%) | 759 | (57.5%) | 0.27 |  |  |
| rs967007 | A | C | 398 | (35.2%) | 734 | (64.8%) | 459 | (34.8%) | 859 | (65.2%) | 0.865 |  |  |

SNP: Single nucleotide polymorphism. Rs number = Reference Single Nucleotide Polymorphism number. ^w^ = Wild type. ^a^ Predicted functional effect on protein synthesis [[44](#_ENREF_44)]. *P-value calculated using Fisher’s exact test. Samples selected for the second stage multivariate analysis in bold. # Odds ratio presented with respect to minor allele > major allele.
