# Supplementary material for: Polymorphisms of SP110 Are Associated with both Pulmonary and Extra-Pulmonary Tuberculosis among the Vietnamese
Source: PLoS One. 2014 Jul 9;9(7):e99496. doi: 10.1371/journal.pone.0099496 (PMC4090157; doi:10.1371/journal.pone.0099496)
Supplement: Table S5 — Allele frequencies for patients with pulmonary TB compared to control subjects. (DOCX) [file pone.0099496.s008.docx]

**Supplementary Table S5:** Allele frequencies for patients with pulmonary TB compared to control subjects.

| *SNP*  *rs number* | *Alleles*  *(Minor:Major)* | | **Allelic frequencies in control subjects** | | | | **Allelic frequencies in pulmonary TB** | | | |  | |  |
| --- | --- | --- | --- | --- | --- | --- | --- | --- | --- | --- | --- | --- | --- |
|  |  |  | *Minor allele* | *(%)* | *Major*  *allele* | *(%)* | *Minor*  *allele* | *(%)* | *Major allele* | *(%)* | *p value** |  |  |
| rs10208770 | G | T | 125 | (11%) | 1007 | (89%) | 155 | (14.7%) | 903 | (85.3%) | 0.013 |  |  |
| rs10498244 | C | T | 165 | (14.6%) | 967 | (85.4%) | 199 | (18.9%) | 853 | (81.1%) | 0.007 |  |  |
| rs1135791 | C | T | 191 | (16.9%) | 941 | (83.1%) | 210 | (19.8%) | 848 | (80.2%) | 0.077 |  |  |
| rs11556887 | T | C | 91 | (8%) | 1041 | (92%) | 75 | (7.1%) | 977 | (92.9%) | 0.467 |  |  |
| rs11678451 | C | T | 165 | (14.6%) | 967 | (85.4%) | 186 | (17.7%) | 866 | (82.3%) | 0.054 |  |  |
| rs1346311 | T | C | 93 | (8.2%) | 1039 | (91.8%) | 91 | (8.6%) | 967 | (91.4%) | 0.758 |  |  |
| rs1365776 | G | A | 113 | (10%) | 1019 | (90%) | 100 | (9.5%) | 958 | (90.5%) | 0.718 |  |  |
| rs1427294 | C | T | 8 | (0.7%) | 1124 | (99.3%) | 2 | (0.2%) | 1050 | (99.8%) | 0.111 |  |  |
| rs16826860 | A | G | 342 | (30.2%) | 790 | (69.8%) | 366 | (34.8%) | 686 | (65.2%) | 0.025 |  |  |
| rs1896258 | G | A | 253 | (22.3%) | 879 | (77.7%) | 211 | (19.9%) | 847 | (80.1%) | 0.174 |  |  |
| rs2114592 | T | C | 241 | (21.3%) | 891 | (78.7%) | 205 | (19.5%) | 847 | (80.5%) | 0.313 |  |  |
| rs2241525 | A | G | 236 | (20.8%) | 896 | (79.2%) | 198 | (18.8%) | 854 | (81.2%) | 0.238 |  |  |
| rs3948464 | T | C | 8 | (0.7%) | 1124 | (99.3%) | 1 | (0.1%) | 1051 | (99.9%) | 0.04 |  |  |
| rs41547617 | A | C | 1132 | (100%) | 0 | (0%) | 1052 | (100%) | 0 | (0%) | na |  |  |
| rs4542839 | C | T | 760 | (100%) | 0 | (0%) | 82 | (100%) | 0 | (0%) | na |  |  |
| rs6436915 | T | G | 429 | (37.9%) | 703 | (62.1%) | 411 | (38.8%) | 647 | (61.2%) | 0.66 |  |  |
| rs6436917 | A | G | 554 | (48.9%) | 578 | (51.1%) | 503 | (47.8%) | 549 | (52.2%) | 0.607 |  |  |
| rs6749579 | G | T | 127 | (11.2%) | 1005 | (88.8%) | 115 | (10.9%) | 937 | (89.1%) | 0.838 |  |  |
| rs7573954 | T | C | 323 | (28.5%) | 809 | (71.5%) | 300 | (28.5%) | 752 | (71.5%) | 1.00 |  |  |
| rs7580900 | C | T | 421 | (37.2%) | 711 | (62.8%) | 395 | (37.3%) | 663 | (62.7%) | 0.965 |  |  |
| rs7601176 | A | G | 129 | (11.4%) | 1003 | (88.6%) | 93 | (8.8%) | 959 | (91.2%) | 0.056 |  |  |
| rs7601299 | A | G | 116 | (10.2%) | 1016 | (89.8%) | 110 | (10.5%) | 942 | (89.5%) | 0.888 |  |  |
| rs919178 | G | A | 507 | (44.8%) | 625 | (55.2%) | 443 | (41.9%) | 615 | (58.1%) | 0.181 |  |  |
| rs967007 | A | C | 398 | (35.2%) | 734 | (64.8%) | 365 | (34.7%) | 687 | (65.3%) | 0.823 |  |  |
